# Remote Patient Monitoring and Digital Therapeutics Enhancing the Continuum of Care in Heart Failure: Nonrandomized Pilot Study

**DOI:** 10.2196/53444

**Published:** 2024-11-06

**Authors:** Emmanuel Marier-Tétrault, Emmanuel Bebawi, Stéphanie Béchard, Philippe Brouillard, Priccila Zuchinali, Emilie Remillard, Zoé Carrier, Loyda Jean-Charles, John Nam Kha Nguyen, Pascale Lehoux, Marie-Pascale Pomey, Paula A B Ribeiro, François Tournoux

**Affiliations:** 1 Faculty of Medicine University of Montreal Montréal, QC Canada; 2 Centre Hospitalier de l’Université de Montréal Montreal, QC Canada; 3 Coeur Research Lab, Research Center of the Centre Hospitalier de l’Université de Montréal Montreal, QC Canada; 4 Department of Management, Evaluation and Health Policy, School of Public Health University of Montreal Montreal, QC Canada; 5 Research Center of the Hospital of Montreal University Montreal, QC Canada

**Keywords:** heart failure, remote patient management, telemonitoring, digital therapeutics, digital health, heart, therapeutics, pilot study, patient care, medical therapy, vitals, weight, symptoms, quality of life, medication optimization, mobile phone

## Abstract

**Background:**

Heart failure (HF) is the primary cause of hospitalization among Canadian patients aged ≥65 years. Care for HF requires regular clinical follow-ups to prevent readmissions and facilitate medical therapy optimization. Multiple barriers lead to therapeutic medical inertia including limited human resources and regional inequities. Remote patient monitoring (RPM) and digital therapeutics (DTx) solutions have been developed to improve HF management, but their adoption remains limited and underexplored. The Continuum project emerged as a collaborative initiative involving a health care center, a software start-up, and an industrial partner.

**Objective:**

We aimed to develop and test the feasibility of the Continuum intervention that seamlessly combined an RPM system with a DTx solution for HF within the same software.

**Methods:**

A 3-month pre-post pilot study was conducted from October 2020 to June 2021. Patients with HF who owned a smartphone or tablet (having remote patient monitoring [RPM+]), had (1) access to a self-care app where they could enter their vital signs, weight, and HF symptoms and view educational content; (2) daily monitoring of their data by a nurse; and (3) a DTx module with automated HF medication suggestions based on national guidelines, made available to their treating medical team. Bluetooth devices were offered to facilitate data recording. Nurses on RPM monitoring could call patients and arrange appointments with their medical team. Patients without a mobile device or unable to use the app were followed in another group (without remote patient monitoring [RPM–]).

**Results:**

In total, 52 patients were enrolled in this study (32 RPM+ and 20 RPM–). Among patients owning a mobile device, only 14% (5/37) could not use the app. In the RPM+ group, 47% (15/32) of the patients used the app for more than 80% (67 days) of the 12-week study period. The use of our digital solution was integrated into the regular nursing workday and only 34 calls had to be made by the nurse during the study period. Only 6% (2/32) of the patients in the RPM+ group experienced at least 1 all-cause hospitalization versus 35% (7/20) of the RPM– ones during the follow-up (6%, 2/32 vs 25%, 5/20 for HF hospitalization) and patients were more likely to have their HF therapy optimized if the DTx solution was available. Quality of life improved in patients compliant with the use of the mobile app (mean score variation +10.6, SD 14.7).

**Conclusions:**

This pilot study demonstrated the feasibility of implementing our digital solution, within the specific context of HF. The seamless integration of Continuum into nursing workflow, mobile app accessibility, and adoption by patients, were the 3 main key learning points of this study. Further investigation is required to assess the potential impacts on hospitalizations, drug optimization, and quality of life.

**Trial Registration:**

ClinicalTrials.gov NCT05377190; https://clinicaltrials.gov/study/NCT05377190 (pilot study #21.403)

## Introduction

Heart failure (HF) is the primary cause of hospitalization among patients aged 65 years or older in Canada [1]. Patients with HF require regular clinical follow-ups to prevent readmissions and to facilitate medical therapy optimization in accordance with national [2] and international guidelines [3,4]. However, numerous barriers hinder the effective implementation of these guidelines, including time constraints and limited human and financial resources, which contribute to therapeutic inertia and recurrent adverse events in this population [5,6].

The COVID-19 pandemic has further exacerbated this phenomenon [7] and forced the rapid development of digital health solutions [2]. Since the onset of the pandemic, telemedicine has emerged as a safe and efficient option for HF follow-up [8]. Many remote patient monitoring (RPM) tools developed specifically for HF are now being implemented in routine clinical practice [9,10]. However, despite the growing adoption of these technologies by health care professionals, evidence of their effectiveness is still limited. The European Society of Cardiology has included RPM in a class 2B recommendation [3], while the American Heart Association has not included it in their latest guidelines [4]. In addition to RPM systems, new tools, best known as Digital Therapeutics (DTx) solutions, have been developed to encourage and facilitate patient medical therapy optimization [11-13], with preliminary data suggesting benefits for patients HF [14].

The Continuum project is a collaborative effort between a health care center, a software start-up, and an industrial partner to develop an intervention that combines an efficient RPM system with a DTx solution for HF. In this study, we present the results from the pilot phase, which aimed to assess the feasibility of implementing this innovative digital solution in a real-world health care environment (technology’s quality, patients’ adoption, and hospital workflow integration). We also explored the potential benefits of the RPM and the DTx solution in our patients with HF with another comorbidity (diabetes).

## Methods

### Study Design

This prospective pilot study was conducted at our HF clinic at the Hospital of the University of Montreal (Centre Hospitalier de l’Université de Montréal [CHUM], Québec, Canada) between October 2020 and June 2021. We enrolled patients with symptomatic HF (New York Heart Association [NYHA] class II to IV), who had diabetes and were 18 years of age or older, regardless of their left ventricular ejection fraction (LVEF). Exclusion criteria were end-stage kidney disease, cirrhosis, cancer with a survival prognosis of less than 1 year, severe cognitive impairment, and patients at high risk of loss of follow-up (ie, history of nonadherence to treatment or substance abuse limiting follow-up appointments). A sample size of 50 patients was intended, as previously described in similar studies [15,16].

A total of 2 frameworks influenced this project the most. The Montreal model was used in this pilot study to properly adapt the solution for patients [17]. Focus groups with patients and caregivers were carried out to gain experiential knowledge in the codesign and development of the software solution in the prevision of adapting the solution before the randomized controlled trial. Also, the Obesity-Related Behavioral Intervention Trials model for developing behavioral treatments for chronic diseases helped us to design and structure this pilot study [6] with behavior change techniques being incorporated into the app development [18,19] (eg, information about HF and self-care tools) [20].

Both RPM and DTx systems were tested during this pilot study. The ability of the patient (or their caregiver) to use the app was assessed by a nurse who was specifically trained for this purpose. After an initial display by the nurse, the patient or their caregiver had 15 minutes to demonstrate their ability to use the app (ie, navigate the app and enter vital signs and symptoms). Patients who did not have a mobile device or did not pass the ability test were followed in the same group (RPM–).

**Figure 1:**
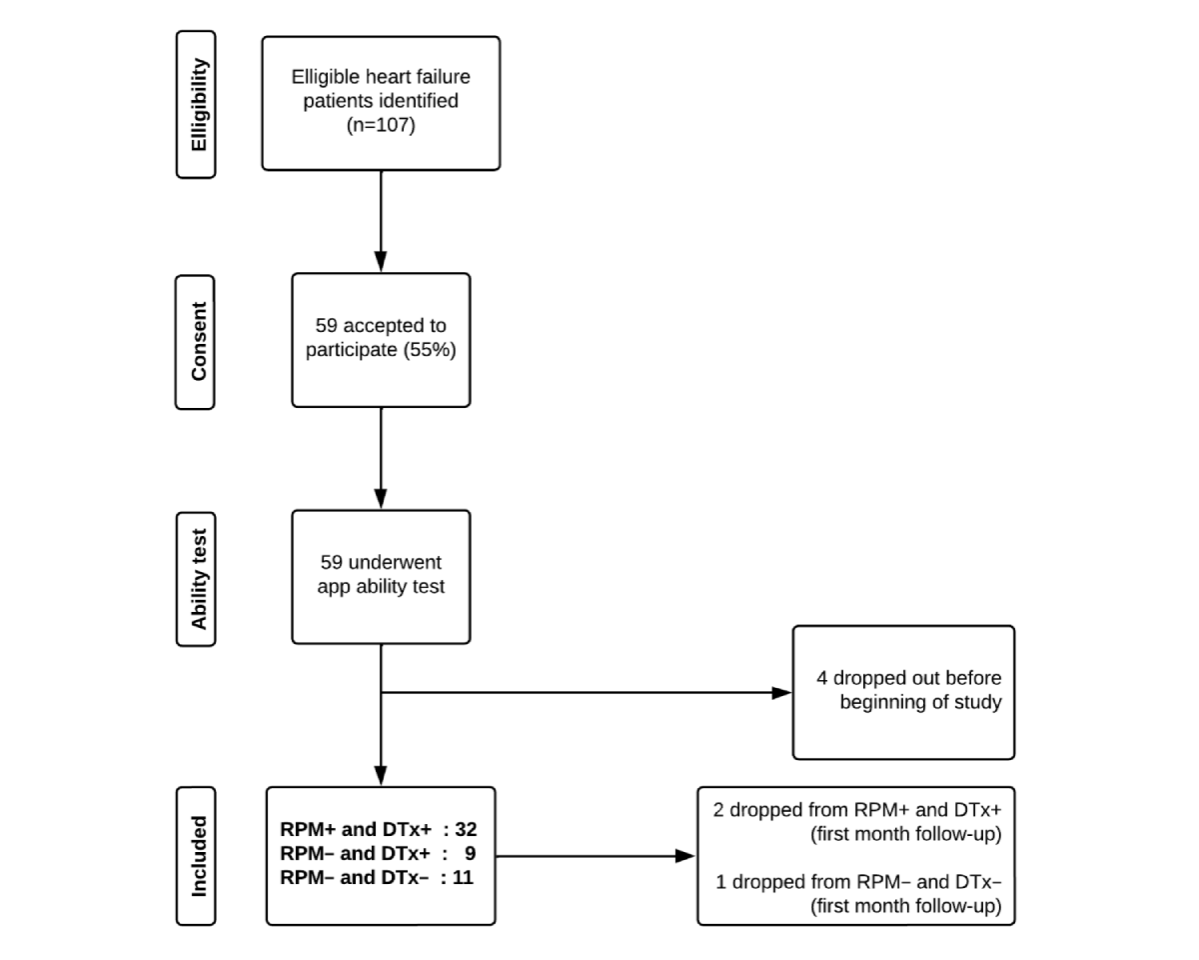
Patient flowchart of the Continuum 3-month pilot study on enhancing heart failure care with remote patient monitoring and digital therapeutics. Eligible patients who accepted (55%) and owned a smartphone or tablet underwent a short ability test. If they passed the test, they were allocated to the RPM+ and DTx+ group. If not, they were randomly assigned into 2 subgroups: RPM− and DTx+ or RPM− and DTx− (with or without DTx medications suggestions). RPM+: having remote patient monitoring; RPM−: without remote patient monitoring; RPM− and DTx+: without remote patient monitoring and having digital therapeutics; RPM− and DTx−: without remote patient monitoring and without digital therapeutics.

All patients in the RPM+ group (intervention group) had their data analyzed by the DTx system, forming the RPM+ and DTx+ group (Figure 1). Patients in the RPM– group were randomly divided into 2 subgroups in a 1:1 ratio. The first subgroup was referred to as RPM– and DTx+, where patients’ data collected at each time point of the study were analyzed by the DTx module even in the absence of the RPM app. The second subgroup was referred to as RPM– and DTx–, where no analysis by the DTx module was performed. This subdivision of the RPM– patients was included to evaluate the impact of using the DTx system alone. In total, 3 groups of patients were followed during this pilot study, namely, RPM+ and DTx+, RPM– and DTx+, and RPM– and DTx–.

Each patient was evaluated at 3 time points: baseline (day 0), 6 weeks, and 12 weeks. Quality of life (QoL) was assessed using the Kansas City Cardiomyopathy Questionnaire-12 (KCCQ-12); hospitalizations and HF medication were recorded at each visit. For patients with LVEF <40%, HF medication was considered as optimized compared with baseline if at least 1 drug class recommended by the Canadian guidelines was added to the patient’s profile or if the dose of at least 1 of the HF drugs was increased during follow-up.

### The Continuum Solution

Each RPM+ patient was offered a package that included 3 Bluetooth-connected devices: a body weight scale, a blood pressure monitor, and a glucometer. However, the use of these devices was not mandatory, and patients had the option to use their own devices if preferred. Technical support was provided to help patients connect these devices to their smartphones or tablets. Patient data, including blood pressure, heart rate, weight, blood glucose level, daily number of steps, and answers to a short survey about HF symptoms, were either automatically transmitted through Bluetooth or manually entered into the mobile app and transferred to the Continuum platform.

To keep patients engaged, specific educational content on HF symptoms, nutrition, physical activity, and mental health was created within the mobile app, and frequent reminders were sent through the app. Once a day, from Monday to Friday, a clinical nurse accessed the web-based dashboard and reviewed the data and alerts triggered by specific algorithms within the Continuum platform. Depending on the type of alert, the nurse could call the patient and conduct a remote visit or arrange for an urgent cardiology consult if needed.

**Figure 2:**
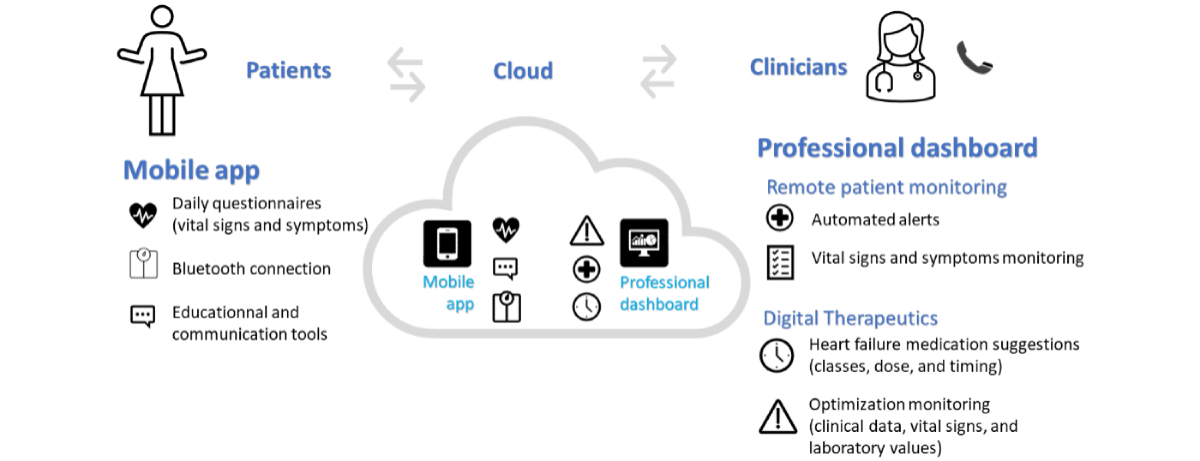
Features of the patient mobile app and health care professional interface used for the Continuum 3-month pilot study using remote patient monitoring and digital therapeutics to enhance heart failure care. Patients transferred their data (vital signs and symptoms) to nurses through a mobile app. Nurses had access to patients’ data through a secured web-based platform and monitored them, once a day (weekdays only). The digital therapeutics guideline-directed medical therapy suggestions were made available on the same platform.

According to the International Organization for Standardization, DTx is “health software intended to treat or alleviate a disease, disorder, condition, or injury by generating and delivering a medical intervention that has a demonstrable positive therapeutic impact on a patient’s health” [21]. Our DTx module was designed to prompt guideline-directed medical therapy (GDMT) optimization according to the latest Canadian HF guidelines; and was integrated into the clinician RPM dashboard [2,22]. Patients’ vital signs (from the mobile app for the RPM+ and DTx+ group or entered manually for the RPM– and DTx+ group), clinical characteristics (NYHA class, LVEF, and comorbidities), current medications, and most recent blood test results were used as inputs. The output consisted of a tailored report that displayed a summary of the patient’s status and suggestions on how HF therapy could be optimized. This report was generated twice, at baseline and 6 weeks, and made available to health care providers, who could use it at their discretion. A summarized scheme of the program is described in Figure 2.

### Data Processing and Analysis

Results are expressed as means (SDs) or as the number of cases and proportions (%), according to allocation groups. The KCCQ-12 score descriptives were performed on patients’ compliance with the use of the application. The scores were classified for their clinical impact according to Spertus et al [23]. Patients were considered as highly compliant if they used the app for >80% of the days during the 12-week study period. For GDMT, the 4 therapeutic classes with a known benefit in HF with a reduced ejection fraction were studied (β-blocker, angiotensin-converting enzyme inhibitor/angiotensin receptor blocker/angiotensin receptor-neprilysin inhibitor, mineralocorticoid receptor antagonist, and sodium-glucose cotransporter-2 inhibitor). GDMT was considered optimized if initiated or uptitrated during follow-up. All analyses were performed using IBM SPSS (version 28).

### Ethical Considerations

The study protocol was approved by the Research Ethics Board of the Centre Hospitalier de l’Université de Montréal (21.403), and all patients provided written consent to participate in the study and secondary data use. Privacy and confidentiality were respected during patient follow-up. Study data were deidentified during the data collection. Data collection, storage, and management were performed using REDCap (Research Electronic Data Capture; Vanderbilt University) [24,25]. No compensation was offered to the participants.

## Results

### Studied Population

A total of 52 patients were enrolled in this pilot study, with 32 in the RPM+ and DTx+ group, 9 in the RPM– and DTx+ group, and 11 in the RPM– and DTx– group (details in Figure 1). Baseline characteristics are detailed in Table 1. Specifically, 71% (37/52) of patients who accepted to participate in the study had a mobile phone and 86% (32/37) were able to use the app appropriately. Most patients (75%) were NYHA class II, and half had LVEF <40%. There were more patients with hypertension and ischemic cardiomyopathy in the RPM– group compared with the RPM+ group. Among the RPM+ patients, 6 out of 32 patients received help from a caregiver to manage the app. Compliance with the app was considered high for 15 out of 32 (47%) patients.

**Table 1:** Baseline characteristics of the heart failure patients enrolled in the Continuum 12-week pilot study. Data are shown for the RPM+ and the RPM– group (the 2 subgroups [RPM– and DTx+; RPM– and DTx–] being combined).

Characteristics			RPM+^a^ (n=32)		RPM–^b^ (n=20)
Age (years), mean (SD)			69.28 (8.9)		73 (11.2)
Sex, female, n (%)			8 (25)		5 (25)
Weight (kg), mean (SD)			88.24 (24.5)		81.4 (17.6)
**NYHA^c^**					
	Class II**,** n (%)	24 (75)		15 (75)	
	Class III**,** n (%)	8 (25)		5 (25)	
Ejection fraction (%), mean (SD)			41.7 (14.5)		43.25 11.3
Ejection fraction ≤40%, n (%)			17 (53)		10 (50)
Ischemic HF^d^, n/n (%)			4/31 (12.9)		7/19 (36.8)
DLP^e^, n (%)			21 (65.6)		15 (75)
HTN^f^, n (%)			20 (62.5)		18 (90)
eGFR^g^ < 60 mL/min, n/n (%)			24/32 (75)		17/19 (89.5)
Hb^h^ < 120 g/L, n/n (%)			12/29 (41.4)		9/16 (56.3)
**NTproBNP^i^**			5384 (9144)^j^		3849 (4117)^j^
	<= 900 ng/L, n/n (%)	9/29 (31.0)		3/19 (15.8)	
	900-1800 ng/L, n/n (%)	6/29 (20.7)		3/19 (15.8)	
	>1800 ng/L, n/n (%)	14/29 (48.3)		13/19 (68.4)	
CIED^k^, n/n (%)			11/32 (34.4)		9/19 (47.4)
**Income brackets (CAD $, n=31)**					
	44,545 (US $33,695), n (%)	22 (71)		18 (90)	
	44,545-66,000 (US $33,695-49,924), n (%)	6 (19.4)		1 (5)	
	66,000-89,080 (US $49,924-67,382), n (%)	3 (10.0)		1 (5)	
Education (years), mean (SD)			13.3 (4.1)		12 (3)
Have a mobile phone or tablet, n (%)			32 (100)		5 (20)

^a^RPM+: having remote patient monitoring.

^b^RPM–: without remote patient monitoring.

^c^NYHA: New York Heart Association dyspnea class.

^d^HF: heart failure.

^e^DLP: dyslipidemia.

^f^HTN: hypertension.

^g^eGFR: estimated glomerular filtration ratio.

^h^Hb: hemoglobin.

^i^NTproBNP: N-terminal-pro-brain natriuretic peptide.

^j^Mean and SD values respectively.

^k^CIED: cardiac implantable electronic device.

### Feasibility

The presentation of the app by the nurse was scheduled for 15 minutes and that time was found to be sufficient according to the clinical team. Nurses in charge of the dashboard could integrate the follow-ups of these patients into their regular workday, usually taking them 15-30 minutes per day to review the dashboard for a cohort of 30 patients. Subsequently, alert management, including calls and remote visits, was performed as needed. A total of 1322 automated alerts were triggered during the entire study, with the majority concerning blood pressure (n=505, 38%) and weight (n=491, 37%), while 158 (12%) were related to glycemia, 118 (9%) to HF symptoms, and 50 (4%) to heart rate values. The same patient could trigger different alerts simultaneously if his or her condition was unstable, for example, experiencing weight gain, worsened dyspnea, and increased edema all at once. Most of these alerts were not clinically significant and were rapidly dispensed with by the nurse. In only 41 situations out of 1322 automated alerts, the nurse had to review the patient’s medical record. The nurse called 34 patients (representing approximately 1 call per patient during the 12 weeks of follow-up), out of which 9 required a medication change, 2 were referred to the emergency room, one was seen as an urgent case at the HF clinic, and one was referred to an electrophysiologist.

### Exploring Potential Clinical Impact of the Continuum Intervention

**Figure 3:**
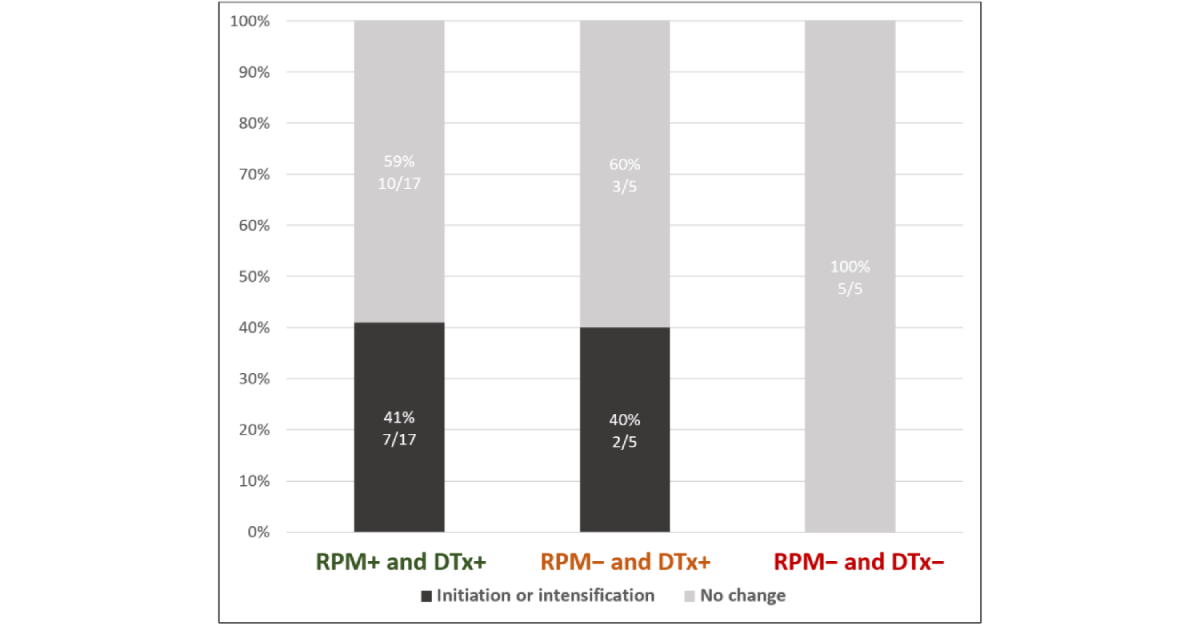
Proportion of patients with left ventricular ejection fraction ≤ 40% having initiation or intensification of heart failure medication (at least 1 class) according to group allocation during the Continuum 3-month pilot study. No initiation or intensification of guideline-directed medical therapies occurred in the RPM− and DTx− in comparison to 40% for the groups with the DTx GDMT suggestions (RPM+ and DTx+; RPM− and DTx+). RPM+ and DTx+: with remote patient monitoring and with digital therapeutics suggestions; RPM− and DTx+: without remote patient monitoring but with digital therapeutics suggestions; RPM− and DTx−: without remote patient monitoring and without digital therapeutics suggestions.

Weight, systolic blood pressure and heart rate were unchanged during the follow-up: 84 (SD 19) kg versus 83 (SD 18), 119 (SD 19) mm Hg versus 117 (SD 18), 71 (SD 9) beats per minute versus 70 (SD 9), respectively. HF GDMT optimization in patients with an ejection fraction <40% only occurred in the RPM+ and DTx+ (7/17, 41%) and in the RPM– and DTx+ (2/5, 40%) groups compared with the RPM–/DTx– (0/5, 0%, Figure 3).

**Figure 4:**
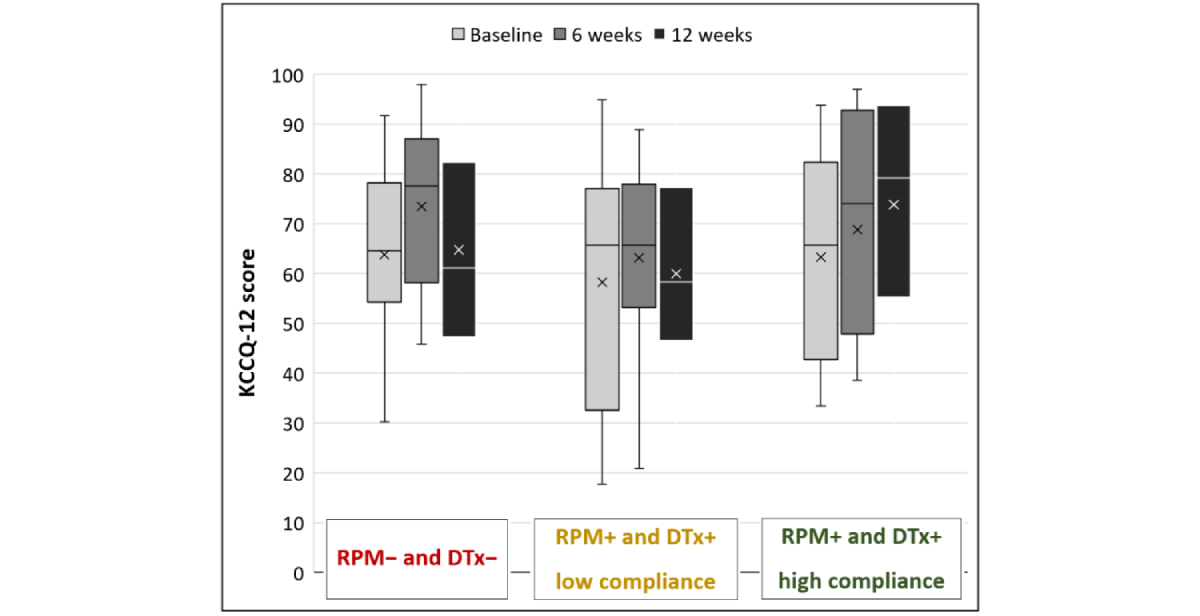
KCCQ -12 Summary Score according to group allocation in the Continuum 3-month pilot study. Groups seem to be comparable in terms of changes in quality of life during the 12 weeks of follow-up between RPM+ and RPM− groups in terms of means and standard deviations. A sub-group showed that highly compliant RPM+ and DTx+ patients had a greater change in quality of life (mean score variation +10.6 (14.7)). KCCQ-12: Kansas City Cardiomyopathy Questionnaire-12; RPM+ and DTx+: with remote patient monitoring and with digital therapeutics suggestions; RPM− and DTx+: without remote patient monitoring but with digital therapeutics suggestions; RPM− and DTx−: without remote patient monitoring and without digital therapeutics suggestions.

Mean variation in QoL during the 12 weeks of follow-up for RPM+ was +5.8 (SD 18.5) versus +0.9 (SD 18.6) for RPM–. A subgroup analysis based on compliance was performed and shown in Figure 4.

**Figure 5:**
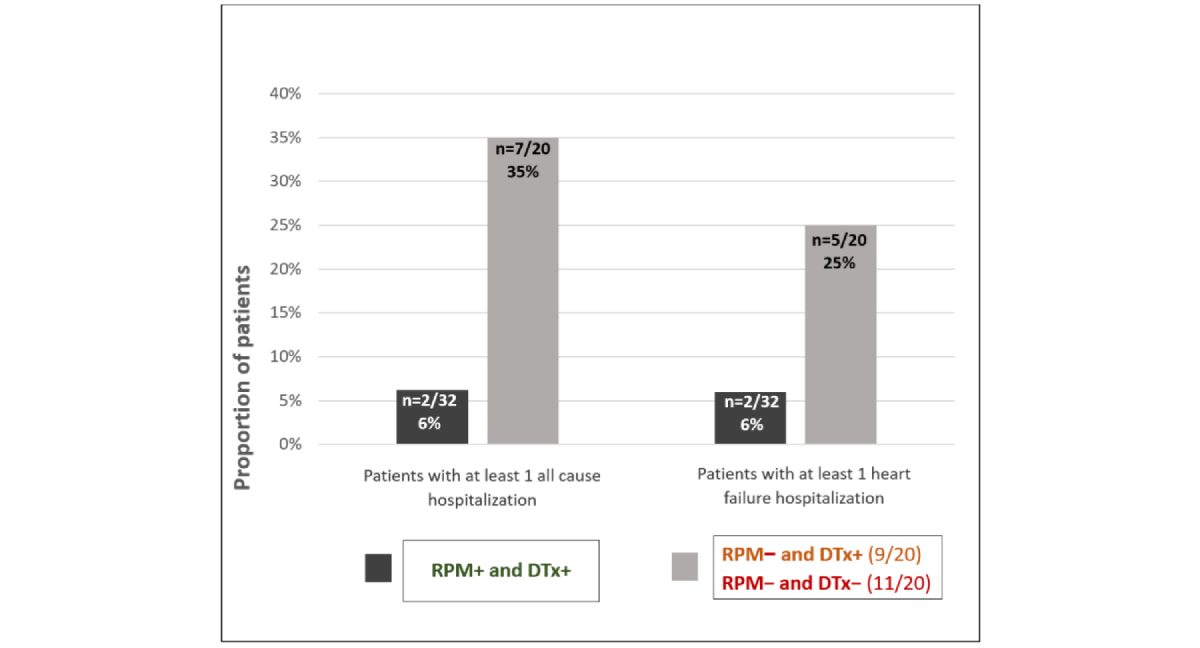
Number of patients with at least one all-cause or heart failure hospitalization according to group allocation during the Continuum pilot study. The number of patients with hospitalizations appears lower in the RPM+ group for both outcomes. RPM+ and DTx+: with remote patient monitoring and with digital therapeutics suggestions; RPM− and DTx+: without remote patient monitoring but with digital therapeutics suggestions; RPM− and DTx−: without remote patient monitoring and without digital therapeutics suggestions.

A total of 35% of the RPM– patients had at least one all-cause hospitalization (Figure 5) during follow-up, with 6% of RPM+ ones. HF-related hospitalizations occurred in 25% of the RPM– patients and 6% in the RPM+ ones.

## Discussion

### Principal Findings

This prospective pilot study gave us insights into the feasibility and potential benefits of implementing a solution using both RPM and DTx systems for patients with HF. Preliminary data suggest a potential favorable impact on hospitalization events, QoL for highly compliant patients, and drug optimization.

### Technology Adoption

An RPM solution should not only try to prevent acute HF decompensation and hospitalization but also improve patient engagement and self-care [9,26]. To ensure future patient acceptance, it is critical to include them as early as possible in the development of the solution [27]. The patient interface of the Continuum solution has been designed with close patient collaboration. This design process was key to patient engagement during the study, 47% of the patients in the intervention group were considered highly compliant, which is consistent with findings from other studies [28].

### Barriers to Implementation

On the patient side, common barriers to telecare implementation include low income, low level of education, living alone, and frailty [29]. However, mobile phone use has more than doubled in the last decade [30] and 60% of Canadians over 65 have a mobile phone now [31]. In our study, 14% (5/37) of our patients who had a mobile phone were unable to use the app properly. In the near future, with the rapid development of more user-friendly interfaces, patients unable currently to use these technologies (who are often older, less educated, and with a lower economic status [32]) should have better access to this type of intervention and inequity in care should be hopefully reduced [33]. For example, patients unable to use their phone because of a visual handicap could benefit from voice-detection and recognition technologies [34] which are well accepted by the patients and easy to integrate into digital health solutions like ours.

On the health care professional side, the massive influx of incoming information is increasingly an obstacle to implementation, substantially burdening clinician workloads [35]. To mitigate this, automation such as thresholds for clinical alerts (ie, blood pressure values, changes in weight, and HF symptoms) should be constantly reassessed and adjusted [35]. The time spent on a telemonitoring platform should be optimized, especially in a context where the cost-effectiveness of telemedicine is uncertain [36]. Similarly to the patient interface, the health care professional interface has been designed with all the stakeholders of a typical multidisciplinary HF team [22], including doctors, nurses, pharmacists, and a psychologist. As expected, nurses focused on how quickly they could examine or check the dashboard every morning to triage which patient’s alert had to be addressed first, while physicians were more interested in how the DTx solution could give them a better understanding of the GDMT optimization suggestions. It is important to consider the needs and challenges of each team member if we want this kind of intervention to succeed.

### The Role of Digital Therapeutics in Heart Failure Guideline-Directed Medical Therapy Optimization

Although GDMT reduces mortality and morbidity [2-4], there remain significant gaps in optimizing GDMT [5]. DTx can be part of the solution since, by definition, they represent various interventions to treat, manage, or prevent a medical disorder or disease [13]. Our DTx interface focused on HF GDMT optimization with the ability to change our algorithm in case of future updates of Canadian HF guidelines [2,22]. In this pilot study, our DTx module seemed to positively influence the overall GDMT profile of these patients but this needs to be confirmed at a larger scale.

### Limitations

This pilot study was not powered and did not have a control group to assess the impact of the Continuum program on patients’ QoL and hospitalization. However, patient-reported outcomes are important in this specific context [37]. Using the KCCQ-12 score in our pilot study, we observed a trend for a positive impact of our intervention on QoL in our most compliant patients, which is consistent with previous reports [38]. An increase of 10.6 points in the KCCQ-12 score in this group is considered a moderate to large clinically significant change [23]. In future work, we will include other patient-reported outcomes such as the European Heart Failure Self-care Behavior questionnaire and the Mobile Application Rating Scale [37].

Similarly to QoL, there was some signal in favor of the RPM+ patients on hospitalization rate but this will be investigated in our next randomized controlled trial (NCT05377190) [39].

Finally, each component of the Continuum program, including the mobile app and RPM and DTx modules, underwent continuous development during the study to incorporate feedback from both patients and health care professionals so the impact of Continuum on patients enrolled at the beginning of the study may have differed from those enrolled later.

### Conclusion

This pilot study demonstrated the feasibility of implementing our digital solution, within the specific context of HF. The seamless integration of Continuum into nursing workflow, mobile app accessibility, and adoption by patients, were the 3 main key learning points of this study. To our knowledge, this is the first combined DTx and RPM solution developed to support clinicians with both HF signs and symptoms monitoring and drug optimization. Further investigation is required to assess the potential impacts on hospitalizations, drug optimization, and QoL.

## Abbreviations

DTx: digital therapeutics
GDMT: guideline-directed medical therapy
HF: heart failure
KCCQ-12: Kansas City Cardiomyopathy Questionnaire-12
LVEF: left ventricular ejection fraction
NYHA: New York Heart Association
QoL: quality of life
REDCap: Research Electronic Data Capture
RPM– and DTx–: without remote patient monitoring and without digital therapeutics
RPM– and DTx+: without remote patient monitoring and having digital therapeutics
RPM: remote patient monitoring
RPM–: without remote patient monitoring
RPM+: having remote patient monitoring

## Data Availability

The data sets generated during and/or analyzed during this study are available from the corresponding author on reasonable request.
